# Stratum-specific health outcome estimation in Pakistan using double goal CART

**DOI:** 10.1371/journal.pone.0294736

**Published:** 2024-02-29

**Authors:** Muhammad Hamza, Shakeel Ahmed

**Affiliations:** National University of Sciences and Technology, Islamabad, Pakistan; Sunway University, MALAYSIA

## Abstract

Post-stratification is applied when the subpopulation membership is observed only for sampled values and the goal is to estimate stratum-specific parameters which leads the survey statisticians towards primary goals i.e., classification of non-sampled units into different strata and prediction of the values of the study variables. Regression models, on one side, optimize the prediction of the study variable’s non-sampled values while the classification algorithms, on the other side, look for the classification of non-sampled cases into different strata. Hence, it is crucial to deal with these two goals simultaneously for the estimation of stratum-specific parameters. This study introduces the idea of a double-objective classification and regression trees (CARTs) approach for estimating stratum-specific parameters. Theoretical properties of the total estimator are derived. An application on the estimation of health outcomes in different domains is given to delineate the practical significance as well as the efficiency of the proposed CART-based method. The proposed estimator of population total performs better than the existing stratum-specific estimator in terms of relative efficiency for all choices of parameters. As an ensemble model, the random forest CART outperforms the other competing tree-based models and homogenous population model without using any auxiliary variable.

## 1. Introduction

Survey statisticians have made major advances to the science of probability sampling, but most practitioners oppose the use of uncontrolled sampling due to the large variation in sampling units. Stratified random sampling controls the diversification with regard to the key study characteristics while maintaining the sample’s probabilistic nature. Numerous studies have been published for the modification and enhancement of the stratification methods following Neyman’s (1938) [1], groundbreaking work. Stratified sampling is justified only when the stratification variable is known prior to the sample selection. However, because such variables fluctuate over time and the sampling frame is built using census data from a few years ago, it is difficult to locate updated information about stratum indicators like house size, socioeconomic status, and education in the majority of health-related surveys.

Post-stratification, on the other hand, refers to the observation of the values of the study variable and the stratum membership variable after the sample has been chosen. For instance, a demographic survey typically can’t stratify according to age, because the age of individuals is not available until the sample is collected. Post-stratification is the practice of using auxiliary data in finite population parameter estimation to improve the precision and accuracy of estimates of the population parameters. [2] uncovered the method of post-stratification that leads to an impressive reduction in the level of sampling to get reasonable estimates of the population. By using a sampling strategy known as multiple inverse sampling [3], attempted to overcome the challenge of post-stratification in high sample sizes, as post-stratification is as effective as ordinary stratification with proportional allocation. Moreover [4], focused on the effects of the post-stratification procedure used in labor force survey (LFS) and investigated whether one can obtain a more precise estimator of parameters using registered information in post-stratification by using auxiliary information or not. Predictive modeling was used to examine post-stratification, with population values assumed to be random variables produced by a model and population quantities inferred using those models’ predictive capabilities [5].

Aside from design-based estimators, model-based approaches rely on the model relationship between the response variable and the predictors for enhancing the precision of estimates. Initially [6], used a straightforward regression model using auxiliary variables to predict the totals of the non-sampled units and their unknown and random quantities. [7,8] predicted the non-sampled values of the study variable in estimating the finite population total using a smooth function. Moreover, [9] introduced a model-based estimator that works with penalized spline regression function to obtain the model-assisted estimator of the total population by using the classical local polynomial regression (CLPR). After that [10], employed a model-based approach to estimate the unknown parameter of the study variable using local linear regression (LLR). Similarly [11], analyzed data in complex surveys by considering the nonparametric estimation methods. Later [12], proposed a novel method for estimating a finite population parameter, which considers a linear combination of population values in a super-population scenario with a known basis function regression (BFR) model. [13] discussed applying linear, mixed, nonparametric, and machine learning techniques to estimate finite population parameters using complex survey data and auxiliary information Under commonly used feature selection criteria in machine learning, the suggested estimator’s prediction error variance was computed. In order to apply machine learning predictions on unobserved data [14], suggested an active sampling technique for data subsampling. By overcoming design constraints, this method enhances performance in virtual simulation-based safety assessment of advanced driver assistance systems. Moreover [15], suggested a method that incorporates data from several sources with accepted practices to get estimates that are precise and reliable. They added that Big Data, which gives more rapid and detailed statistics, offers a solution to diminishing response rates and survey expenses. To make the transition from intended data to data-oriented statistics, it is necessary to comprehend the prerequisites for reliable inference. This goal is concretized through a number of statistical frameworks, however, these are broad approaches.

In machine learning, tree-based methods are favored due to ease of application and capturing linear and/or non-linear relationships between the variables without assuming a specific functional trend. Classification and Regression Tree (CART) algorithm to predict target variable values based on covariates and provide easily interpretable results. CARTs perform categorization and prediction based on observed data and can be employed for predicting the values of unobserved data. [16] examined the prediction performance of decision trees like CART and compared the results with those of other tree-based methods. Further [17], studied the prediction performance of decision trees like CART and a comparison has been also made between various tree-based algorithms. The performance of three non-parametric tree-based approaches was later examined by [18], for general forest mapping with high-resolution SPOT-HRG data because traditional methods like field surveys are time- and money-consuming. After that, [19] examined the effectiveness of software defect prediction as a research area in software engineering and the prediction capabilities of seven tree-based ensembles. Similarly [20], stated that the decision tree algorithm is the most important and efficient machine learning method.

[21] investigated the method of automatic diabetes prediction using random forest and gradient boosting classifiers. These tree-based ensemble methods with proper data processing, hyper-parameter tuning, and oversampling, can effectuate above 90% accuracy. Recently [22], developed a model-assisted technique based on random forests and estimated the functional relationship between the survey variable and the auxiliary variables. They also established the theoretical features of the procedure and calculated the associated estimator. Additionally, a model calibration process for dealing with numerous survey variables was covered.

When we need separate estimates in different study domains model-based approaches may be used for two purposes. First, the model is used for the classification of units in different study domains and secondly, a specific model will be applied for the prediction of non-sampled values.

The main contribution of the paper is to use two tree-based machine learning algorithms for obtaining separate estimates in different sup-population called domains, where the domain membership is observable only in the sample. The method proposed for the estimation of finite population parameters (population total) in this study uses a classification tree-based algorithm for classifying non-sampled units (the units not selected in the sample) into different strata (domains) and a regression tree-based algorithm for prediction of the values of the study variable on non-sampled units. To evaluate the performance of the estimator suggested in this study and to assess the applicability of the method we use bootstrap studies for two situations taking different health-related variables as the variables of interest.

In Section 2, we provide an overview of the classical model-based stratum-specific total estimator of population total with its finite sample properties. Section 3 provides the proposed tree-based algorithm for estimating stratum-specific total. Section 4 comprises bootstrap studies for two different cases to evaluate the performance of a stratum-specific total estimator. Section 5 concludes the study with some future recommendations.

## 2. Existing model-based estimation method

Let *U* = {1,2,3,….,*N*} be the set of serial number attached to the units in a finite population of size *N*. Further *Y* and *X* be the study and auxiliary variables with values *y*_*i*_ and *x*_*i*_ corresponding to the *ith* population unit for all *i* ∈ *U*. The population consists of *H* mutually exhaustive strata whose membership are assumed to be unknown prior to the survey. The stratum membership variable for the ℎ^*t*ℎ^ stratum can be defined as *A*_*hi*_ which possess value *a*_*hi*_ = 1 if *i*^*t*ℎ^ unit belongs to ℎ^*t*ℎ^ stratum, *a*_*hi*_ = 0 otherwise, such that

$$y_{i}=\mu_{h}+\epsilon_{hi}$$ (1)

fori=1,2,3,…..,Nandh=1,2,…..,H,

The stratum membership variable *A*_*hi*_ for *h* = 1,2,…,*H* is defined as independently distributed Bernoulli random variables. The mean and variance of the product *A*_*h*_*Y* can be obtained as

$$E_{M}(AY)=E_{M}(Y_{i}|A_{hi}=1)p(A_{hi}=1)=\mu_{h}\lambda_{h},$$ (2)

and

$$V_{M}(A_{Y})=\lambda_{h}\sigma_{h}^{2}+\lambda_{h}(1-\lambda )\mu_{h}^{2}=\sigma_{h}^{*2}(say)$$ (3)

The covariance between *A*_*hi*_*Y*_*i*_ and *A*_*hi*_*Y*_*j*_ for *i*≠*j*∀*i*,*j*∈*U* is zero as *Y*_*i*_ (conditionally) and *A*_*hi*_ both are independent random variables. Following Chamber and Clark (2012) [23], the expansion estimator for the ℎ^*t*ℎ^ stratum total ${\hat{t}}_{yh}$, is given by:

$$t_{yh}^{E}=\frac{N}{n}\sum a_{hi}y_{i}=N\;{\hat{\lambda }}_{h}{\bar{y}}_{h}$$ (4)

where ${\bar{y}}_{h}=\frac{\sum_{i=s}a_{hi}y_{i}}{n}$ is the sample mean for the *hth* stratum and ${\hat{\lambda }}_{h}=\frac{n_{h}}{n}$ is the estimator of *λ*_*h*_. The derivation of ${\hat{t}}_{yh}^{E}$, after taking expectation of the prediction error, is given by

$$Bias_{M}({\hat{t}}_{yh}^{E})=\lambda_{h}\mu_{h}\left[\sum_{i\in s}w_{hi}^{*}-(N-n)\right].$$ (5)

with the unbiasedness condition

$$\sum_{i\in s}w_{hi}^{*}=(N-n).$$

The model variance of the prediction error can be obtained as

$$V_{M}({\hat{t}}_{yh}^{E}-t_{yh})=\sum_{i\in s}w_{hi}^{*2}V_{M}(a_{hi}y_{i})+\sum_{i\in s}V_{M}(a_{hi}y_{i})-2Cov\left(\sum_{i\in s}w_{hi}^{*}(a_{hi}y_{i}),\sum_{i\in s}\left(a_{hi}y_{i}\right)\right)$$

$$V_{M}({\hat{t}}_{yh}^{E}-t_{yh})=\lambda_{h}^{*2}[\sum_{i\in s}w_{hi}^{*2}+(N-n)]$$ (6)

The expansion estimator ${\hat{t}}_{yh}^{E}=\frac{N}{n}\sum_{i\in s}a_{hi}\;y_{i}$ with variance

$$V_{M}({\hat{t}}_{yh}^{E}-t_{yh})=\sigma_{h}^{*2}\left[\frac{{\left(N-n\right)}^{2}}{n}+(N-n)\right]=\frac{N(N-n)}{n}\sigma_{h}^{*2}.$$ (7)

The expansion estimator ${\hat{t}}_{yh}^{E}$ has two attractive features one is BLUP property with respect to the model and the other is the compensation for unknown stratum size. However, the expansion estimator does not consider the known auxiliary information. However, classical model-based estimation approaches with auxiliary variables have filled this gap (see, [13]). When the model relationship between the study variable and the auxiliary variables is non-linear, we can no longer rely on the classical model-based estimators. The tree-based estimation procedure fills this gap and aids in efficiency improvement for the estimation of finite population parameter estimation.

## 3. Proposed model-based estimation method

**Fig 1:**
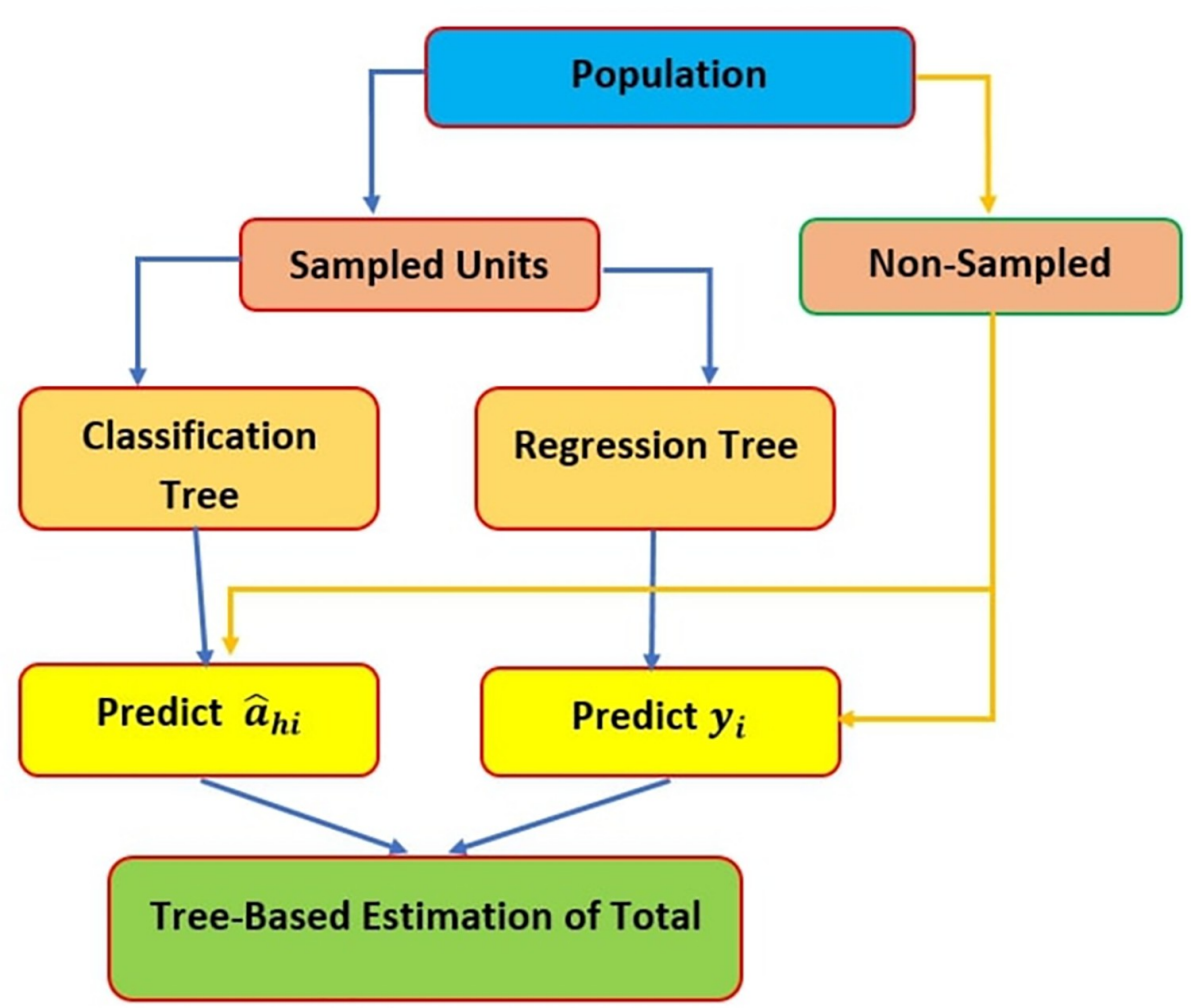
Flow diagram of DGCART.

Decision trees are non-parametric methods to screen the data into meager, extra “pure or homogenous groups known as nodes. An easy way to define “purity” is by increasing accuracy or by decreasing misclassification error. Decision tree models are suitable when there is a good reason to suspect non-additive interaction among variables or there are far too many variables under study. In general, depending on whether a statement is true or untrue, a decision tree will make a statement. CART is better at detecting this relationship than the use of interaction terms in linear models. Tree-based methods are favored for ease of application, captures linear and/or non-linear relationship between the variables without assuming a specific functional trend, and do not assume that all study variables are equal. To categorize the non-sampled units into strata and to predict the values of the study variable for the non-sampled part, two tree-based methods are used simultaneously and called it the double-goal classification and regression tree (DGCART) approach. Here, we modify the CART method to fulfil the dual objectives of stratification and prediction. The DGCART-based estimation algorithm is summarized in Table 1 and Fig 1.

**Table 1:** Illustration of DGCART for estimation of finite population total.

*Step*	*Description*
***1*.**	Select a simple random sample of size *n* leaving *N* − *n* units as non-sampled from the population U.
***2*.**	Observe the study variable *Y*, and the domain membership variable *a*_*h*_ (*h* = 1,2,3,…..,*H*). Utilize Attribute Selection Measure (ASM) to identify the dataset’s top attributes. The Gini index is used as the measure of impurity or purity used to construct a decision tree. Finding the attribute that yields the most information gain is the key for building a decision tree.
***3*.**	Grow a classification tree from the sampled data i.e., [*a*_*h*_: *x*1, *x*2, *x*3… *x*_*p*_] for *hth* stratum.
***4*.**	Classify the non-sampled units according to the tree grown in step 2.
***5*.**	Grow a regression tree from the sampled data [*Y*: *x*1, *x*2, *x*3… *x*_*p*_] for prediction of the study variable.
***6*.**	Predict the non-sampled units according to the tree grown in step 4. An illustration for tree-based estimation of parameters in domain. The illustration is given in Fig 1.

We construct classification trees for the sampled data in Step 2, as a result, we obtained ′*L*′ nodes numbered as 1,2,…,*l*,…,*L* representing by a set of classes *C* = {*C*_1_, *C*_2_,…,*C*_*l*_,…,*C*_*L*_}. We then classify the nodes into different strata according to the majority vote i.e., the *lth* node will be classified to *hth* stratum if *n*_*hl*_ = max {*n*_1*l*_, *n*_2*l*_,…,*n*_*Hl*_}.

The stopping rule for the classification tree is made by observing the increase in variance of *A*_*h*_(*h* = 1,2,…,*H*). We set a reduction in variation function as

$$\Delta \{V(A_{h}|C_{F},C_{F-1})\}=V(A_{h}|C_{F})-V(A_{h}|C_{F-1})$$ (8)

where $E(A_{h}|C_{F})=\lambda_{h},V(A_{h}|C_{F})=\lambda_{h}(1-\lambda_{h}),$ *C*_*F*_ is the set of classes at final node of classification tree and *C*_*F*−1_ is the set of classes at the node proceeding to the final node of the classification tree. At final node the *λ*_*h*_ is estimated using the node specific data. i.e.

$${\hat{\lambda }}_{h}=\frac{\sum_{C_{F}}a_{hi}}{n_{F}},$$ (9)

where *n*_*F*_ is the total number of units at the final node $n_{F}=\sum_{C_{F}}n_{l}$.

The process continues until $\hat{\Delta }\{V(A_{h}|C_{F},C_{F-1})\}$ does not fall below a pre-specified value Δ_*o*_. At this stage one should ensure that the sample size at note *t* for a given *h* should be at least 2 i.e., *n*_*th*_≥2. Once the stopping criterion is met, we get the classified data as *C* = {*C*_1_, *C*_2_,…,*C*_*l*_,…,*C*_*L*_}. After classification of non-sampled units into different domains, we grow a regression tree from the sampled data i.e., [*y*: *x*_1_, *x*_2_, *x*_3_,…,*x*_*p*_] for prediction of the values of study variable.

Moreover, let $C^{*}=\{C_{1}^{*},C_{2}^{*},\ldots ,C_{t}^{*},\ldots ,C_{T}^{*}\}$ be the set of nodes on regression tree constructed on sampled units. The values of the non-sampled units are predicted as the mean of the sampled values on a given node for example at *t*^*th*^ node the value of *y*_*i*_ is predicted as:

$${\bar{y}}_{t}=\frac{\sum_{i\in C_{t}^{*}}y_{i}}{n_{t}},$$ (10)

The stopping rule is made for regression tree by observing the increase in variance of the study variable *y* at given node. We set a reduction in variation function as:

$$\Delta \{V(Y_{i}|C_{F}^{*},C_{F-1}^{*})\}=V(Y_{i}|C_{F}^{*})-V(Y_{i}|C_{F-1}^{*})$$ (11)

where $E(Y_{i}|C_{F}^{*})=\mu_{y},V(Y_{i}|C_{F}^{*})=\sigma_{y}^{2},\;C_{F}^{*}$ is the set of classes at final node of regression tree and $C_{F-1}^{*}$ is the set classes at the node proceeding to the final node of the regression tree. At final node, the mean, and the variance of is estimated using the node specific data for *h*^*th*^ domain as:

$${\hat{\mu }}_{yh}=\bar{y_{i}},\;\mathrm{and}\;{\hat{\sigma }}_{yh}^{2}=\frac{\sum_{C_{F}^{*}}a_{hi}{\left(y_{i}-{\bar{y}}_{t}\right)}^{2}}{n_{F}-1}$$

The predictive estimation problem starts with partitioning the total of *h*^*th*^ stratum into sampled and non-sampled parts. The *i*^*th*^ value of the study variable for non-sampled part is, then, predicted using the mean value of that class. The resulting tree-based estimator for domain total is given by

$${\hat{T}}_{tbh}=\sum_{s}a_{hi}\;y_{i}+\sum_{\bar{s}}{\hat{a}}_{hi}\;{\hat{y}}_{i}$$ (12)

$${\hat{a}}_{hi}=\left\{ \begin{array}{c} 1\;\mathrm{for}\;C_{l}\;when\;n_{hl}\;is\;\mathrm{maximum}\\ 0\;\mathrm{elsewhere} \end{array}\right.\;\mathrm{for}\;l=1,2,\ldots L$$

and ${\hat{y}}_{i}=\frac{\sum_{C_{F}^{*}}y_{i}}{n_{T}}$, after some simplification, we have

$${\hat{T}}_{tbh}=\sum_{i\in s}a_{h}y_{i}+r_{h}{\bar{Y}}_{hC_{t}^{*}}$$ (13)

where ${\bar{Y}}_{hC_{t}^{*}}=\frac{\sum_{i\in \bar{s}}{\hat{a}}_{hi}{\bar{y}}_{C_{t}^{*}}}{r_{h}}{,\;\hat{r}}_{h}=\sum_{i\in \bar{s}}\;\sum_{l=1}^{L}{\hat{a}}_{hi}r_{l},\;r=\sum_{l\in 1}^{L}r_{l},\;and\;n_{h}=\sum_{i\in \bar{s}}\;\sum_{l=1}^{L}{\hat{a}}_{hi}n_{l}$.

An estimate of *r*_*h*_ can be obtained as:

$${\hat{r}}_{h}={\hat{\lambda }}_{h}^{*}r$$ (14)

Inserting estimated value of ${\hat{r}}_{h}$ in (13), we get

$${\hat{T}}_{tbh}=n{\hat{\lambda }}_{h}{\bar{y}}_{sh}+{\hat{\lambda }}_{h}r{\bar{y}}_{hC_{t}^{*}}$$

$${\hat{T}}_{tbh}=n\;{\hat{\lambda }}_{h}({\bar{y}}_{sh}-{\bar{y}}_{hc_{t}^{*}})+{\hat{\lambda }}_{h}N\;{\bar{y}}_{hC_{t}^{*}}$$ (15)

When classification does not divide the data in a meaningful way the combined stratum mean ${\bar{y}}_{hC_{t}^{*}}$ coincide with the overall stratum specific mean ${\bar{y}}_{sh}$ i.e., ${\bar{y}}_{hc_{t}^{*}}={\bar{y}}_{sh}$ and, as a result, the tree-based total estimator gives similar result as the expansion estimator, i.e. ${\hat{T}}_{yh}={\hat{\lambda }}_{h}N{\bar{y}}_{sh}$. The prediction error of the tree-based total estimator can be written as

$${\hat{T}}_{tbh}-T_{yh}=\sum_{i\in \bar{s}}{\hat{a}}_{hi}\;{\bar{y}}_{t}-\sum_{i\in \bar{s}}\;a_{hi}y_{i}$$ (16)

Applying model expectation on Eq (16), the prediction error given the set of classes *C*_*l*_ is

$$E({\hat{T}}_{tbh}-T_{yh}|C_{l},a_{hi}^{*}=1)=\sum_{i\in \bar{s}}E(a_{hi}^{*}{\bar{y}}_{t})-\sum_{i\in \bar{s}}E(a_{hi}y_{i}).$$ (17)

we have

$${E(\hat{a}}_{hi}\bar{y_{t}}|C_{l},a_{hi}^{*}=1)=E\{E(a_{hi}{\bar{y}}_{t}|a_{hi}^{*}=1)\}=E\{a_{hi}^{*}\mu_{lh}\}=\mu_{lh}{\lambda^{*}}_{h}$$

Inserting ${E(\hat{a}}_{hi}\bar{y_{t}}|C_{l},a_{hi}^{*}=1)$ in Eq (17), we get the conditional bias as follow

$$B({\hat{T}}_{tbh}-T_{yh}|C_{l},a_{hi}^{*}=1)=(N-n)(\lambda_{h}^{*}\sum_{l=1}^{L}\mu_{lh}-\mu_{h}\lambda_{h})$$ (18)

The Model bias term reduces as the sum of class specific mean reaches to overall population mean which is the worst situation in terms of efficiency. To increase in efficiency, we need to compromise some amount of bias in prediction process.

Similarly

$$V({{\hat{a}}^{*}}_{hi}{\bar{y}}_{t}|C_{l})=V\{E({{\hat{a}}^{*}}_{hi}{\bar{y}}_{t}|{{\hat{a}}^{*}}_{hi}=1,C_{l},a_{hi}^{*}=1)\}+E\{V({{\hat{a}}^{*}}_{hi}\bar{y_{t}}|{{\hat{a}}^{*}}_{hi}=1,C_{l})\}={{\lambda^{*}}_{h}{\sigma^{*2}}_{t}+\mu }_{lh}{\lambda^{*}}_{h}(1-{\lambda^{*}}_{h})$$ (19)

Further, Variance of bias of ${\hat{T}}_{tbh}$ is given as:

$$V({\hat{T}}_{Tbh}-T_{yh})=\sum_{s}V({\hat{a}}_{h}{\bar{y}}_{i}C_{l})+\sum_{\bar{s}}V(a_{h}y_{i})=(N-n)[\{\lambda_{h}^{*}\sigma_{t}^{*2}+\mu_{lh}\lambda_{h}^{*}(1-\lambda_{h}^{*})\}+\{\lambda_{h}\sigma_{h}^{2}+\lambda_{h}(1-\lambda_{h})\mu_{h}^{2}\}]$$ (20)

## 4. Bootstrap studies

We conduct a bootstrapped study using Pakistan maternal mortality survey dataset, 2019 [24], for the two cases: (1) Taking the pregnancy loses as the study variable, and (2) Taking the delivery duration as the study variable. The dataset consists of *N* = 634 observations, after omitting rows having missing responses, with 28 variables (see details of the variables in Appendix A). Considering this dataset as the population, a simple random sample of size (*n* = 20,30,40,50,65, and 75) is drawn. Two separate trees are grown one for prediction problem and the other for classification of the non-sampled units using 5 different CART models and a random forest model. The summary of models used in this study are dscribed in Table 2.

**Table 2:** Details of DGCART Models used in the study (hyper-parameters).

Model	Model’s Type	Min Split	Max Depth	Min Bucket	Cp	No. of Trees
**1**	**Classification**	2	10	0.67	0.001	1
	**Regression**	2	5	0.67	001	1
**2**	**Classification**	3	None	1	0.005	1
	**Regression**	2	10	0.67	0.001	1
**3**	**Classification**	1	None	2	0.003	1
	**Regression**	20	None	1	0.001	1
**4**	**Classification**	20	None	1	0.001	1
	**Regression**	2	6	1	0.003	1
**5**	**Classification**	5	None	6	0.01	1
	**Regression**	3	6	1	0.005	1
**RF**	**Classification**	20	None	1	0.001	500
	**Regression**	20	None	7	0.01	500

We have used different decision tree tune parameters (hyper-parameters) to tune the tree. There are 5 different CART models having different values of hyper-parameters and one random forest model. Different tree parameters including the maximum depth which intended to prevent overfitting the specifics minimal number of observations needed in a node for split to be attempted is specified by “min split” and the “min bucket” (number of observations that are permitted in a terminal node) The value "None" indicates that we didn’t utilize any values for the relevant hyper parameters in the model.

The Expected absolute prediction error (EAPE) of the stratum-specific total estimator is obtained under different models i.e. *k* = 1,2,3,4,5, *rf*.

$$EAPE=\frac{1}{Q}\sum_{j=1}^{Q}|\frac{t_{yh(k)}^{j}-T_{yh}}{N_{h}}|,$$ (21)

where *h* = 1,2, *and Q* denotes the number of simulations. Further, the mean square prediction error (MSPE) of the stratum-specific total estimator is obtained under different models as follow

$$MSPE=\frac{1}{Q}\sum_{j=1}^{Q}{\left(\frac{t_{yh(k)}^{j}-T_{yh}}{N_{h}}\right)}^{2},$$ (22)

where *h* = 1,2, *and Q* denotes the number of simulation.

In R simple tree-based algorithms with some choices of tree size, splitting criteria, the number of trees to be produced, etc. are obtained using rpart package. The rpart employs a metric, like other partitioning algorithms, to choose the optimum rule for dividing the data. The method uses the Gini coefficient as the computational metric.

### Case 1

Comparing Pakistan to other South Asian nations, Pakistan has the highest rate of pregnancy losses (30.6 pregnancy losses per 1000 total births) [25]. There is a paucity of literature on the lived experiences of Pakistani women who have experienced multiple stillbirths, despite the well-documented psychological effects of stillbirths on bereaved women [26]. Multiple stillbirths have a severe effect on women’s emotional and social welfare, so in Case 1, the usage of contraceptive methods has been taken as the stratum membership variable, *h* = 1,2, (Stratum 1 and 2) and the number of pregnancy losses (which ranges in 1 to 20) has been taken as the study variable.

Table 3 shows bootstrap study results for Case 1. There are five different CART models according to tree parameters and one random forest (*rf*) model. The table provides ${\hat{\lambda }}_{h}$ which shows estimated the *hth* stratum proportion. The tables include ${\hat{\mu }}_{yh}$ the mean of respective stratum, the expected absolute prediction error (EAPE), mean square prediction error (MSPE) and relative efficiency (RE) of the estimators for different choices of sample sizes. Table 3 provides that the mean number of miscarriages is higher for Stratum 2 i.e., mothers who have ever used contraceptive methods against women who have not used any birth control. The mean pregnancy losses for the mothers who ever used contraceptive method is in the range [1.5299 to 1.5745] and for those who do not use contraceptive method is [1.3895, 1.4301]

**Table 3:** Bootstrap results for Case 1.

		Stratum 1					Stratum 2				
n	Models	$\hat{\lambda_{h}}$	${\hat{\mu }}_{yh}$	EAPE	MSPE	RE	$\hat{\lambda_{h}}$	${\hat{\mu }}_{yh}$	EAPE	MSPE	RE
**50**	**1**	0.3296	1.4301	0.1630	0.1278	1.1128	0.6711	1.5472	0.0188	0.0495	1.1532
	**2**	0.3295	1.4225	0.1706	0.1276	1.1143	0.6711	1.5724	0.0063	0.0502	1.1363
	**3**	0.3338	1.4219	0.1712	0.1277	1.1135	0.6654	1.5709	0.0047	0.0498	1.1459
	**4**	0.3296	1.4178	0.1752	0.1279	1.1128	0.6711	1.5680	0.0019	0.0495	1.1545
	**5**	0.3338	1.4175	0.1755	0.1280	1.1122	0.6654	1.5669	0.0007	0.0491	1.1626
	**rf**	0.3060	1.4132	0.1798	0.1175	1.2102	0.6904	1.5244	0.0417	0.0364	1.5663
**65**	**1**	0.3308	1.4151	0.2515	0.1424	1.0495	0.6702	1.5499	0.0286	0.0426	1.2717
	**2**	0.3307	1.4152	0.2514	0.1416	1.0556	0.6701	1.5783	0.0003	0.0435	1.2467
	**3**	0.3344	1.4149	0.2516	0.1415	1.0557	0.6650	1.5768	0.0018	0.0429	1.2631
	**4**	0.3308	1.4083	0.2583	0.1434	1.0420	0.6701	1.5707	0.0079	0.0422	1.2838
	**5**	0.3344	1.4084	0.2582	0.1433	1.0428	0.6650	1.5697	0.0088	0.0418	1.2972
	**rf**	0.3168	1.4010	0.2656	0.1368	1.0918	0.6777	1.5308	0.0477	0.0325	1.6681
**75**	**1**	0.3304	1.3992	0.2338	0.1189	1.0151	0.6690	1.5417	0.0582	0.0354	1.4112
	**2**	0.3300	1.4058	0.2273	0.1164	1.0368	0.6691	1.5747	0.0254	0.0350	1.4287
	**3**	0.3332	1.4075	0.2256	0.1165	1.0363	0.6644	1.5726	0.0273	0.0347	1.4380
	**4**	0.3304	1.3952	0.2378	0.1190	1.0145	0.6690	1.5631	0.0368	0.0344	1.4526
	**5**	0.3332	1.3969	0.2362	0.1190	1.0146	0.6644	1.5615	0.0384	0.0342	1.4619
	**rf**	0.3279	1.3895	0.2435	0.1133	1.0651	0.6673	1.5299	0.0700	0.0286	1.7469

Decision trees are non-parametric methods to screen the data into meager, extra “pure, or homogenous groups known as nodes. An easy way to define “purity” is by increasing accuracy or by decreasing misclassification error. Decision tree models are suitable when there is a good reason to suspect non-additive interaction among variables or there are far too many variables under study. The average absolute deviation of the predictions from the true values of the parameter is obtained using the expected absolute prediction error (EAPE) measure for different models. No significant change is observed in EAPE values with a change in tree parameters, however, EAPE values have a slightly increasing trend with an increase in sample size which shows a trend of unbiasedness for larger sample sizes. Further, EAPE values are higher Stratum 1 as compared to Stratum 1.

**Fig 2:**
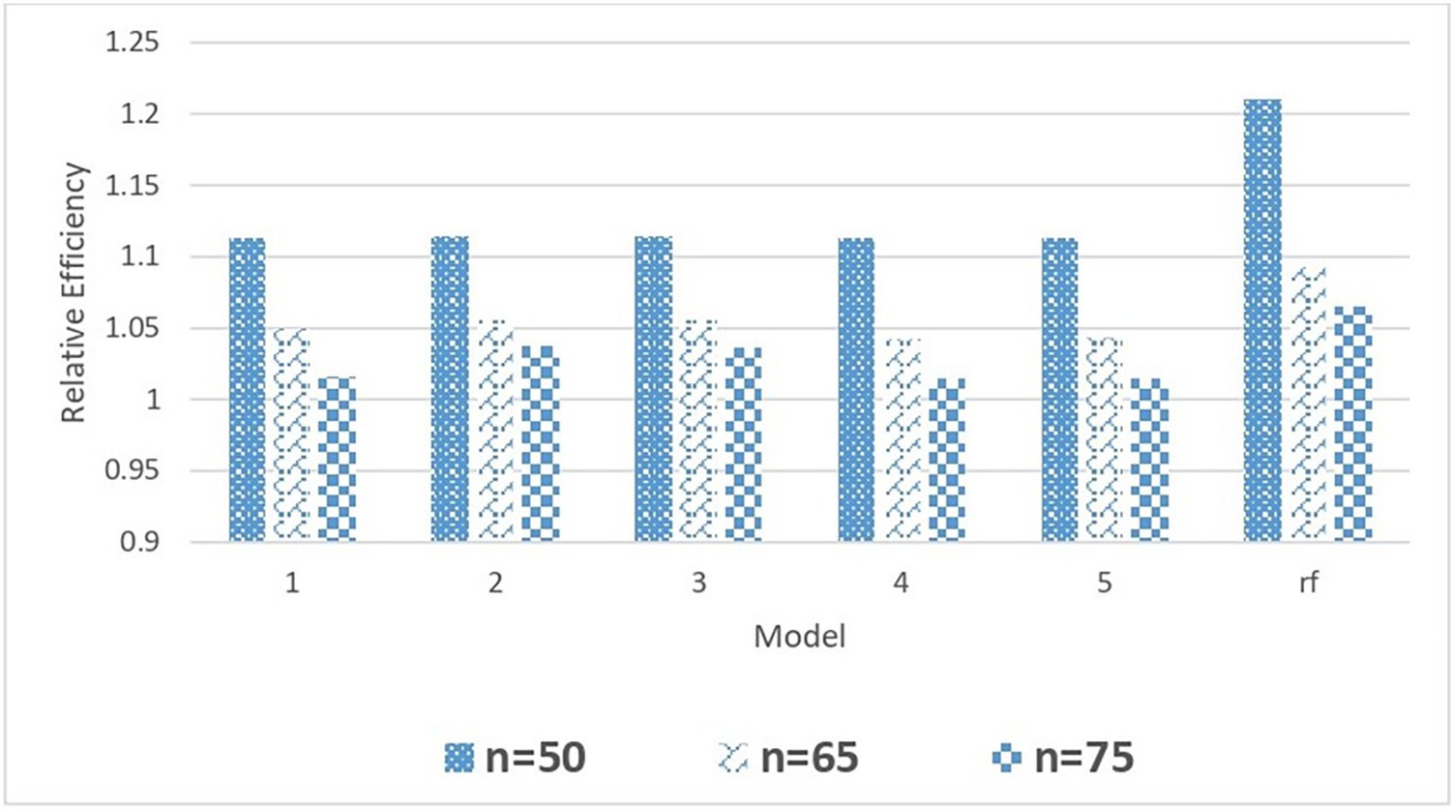
Comparison of relative efficiency of the mean estimator with different DGCART models for different sample sizes under Case 1.

**Fig 3:**
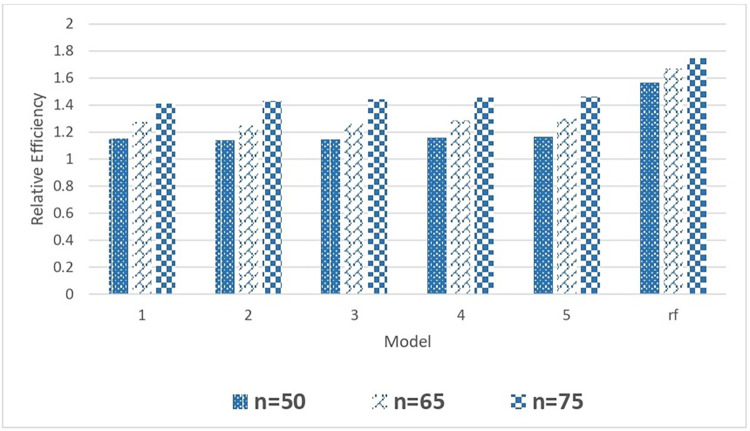
Comparison of relative efficiency of the mean estimator with different DGCART models for different sample sizes under Case 1.

Similar to EAPE, there is no significant change in MSPE values with a change in tree parameters. However, the relative MSPE values corresponding to the random forest is significantly smaller than all other single-tree models. The relative efficiency (RE) values are greater than one for all combinations of tree parameters showing the superiority of tree-based total estimators to corresponding estimators under the homogenous model. However, the value RE of the random forest model is higher among all competing models due to the ensemble technique applied in random forest (rf) algorithms for building classification t and regression models for observed data. The comparison of different competing models used in this study is visually displayed in Figs 2 and 3 for Case 1 bootstrap study.

Fig 2 graphically compares the relative efficacy of five single tree models and one random forest model. With n = 50, all single CART models exhibit comparable relative efficiency. Every model has a different relative efficiency for single CART models with n = 65, but models 2 and 3 have better relative efficiencies. According to the models, relative efficiency for n = 75 has changed, with model 4 having lower efficiency. The relative effectiveness of random forest is higher than that of the other individual CART models for all samples.

Fig 3 compares the relative efficacy of CART models with various sample sizes for the number of miscarriages per woman who did not use any kind of contraception before or throughout her pregnancy. By comparing the relative effectiveness of models with n = 75 to models with n = 50 and n = 65 in single CART models, we may determine that larger sample sizes can yield more accurate population estimates. The relative efficiency for all single CART models is about the same for n = 50, and it is even the same for n = 65. We have superior relative efficiency in the random forest model as compared to other single CART models because random forests build cumulative decision trees. The proposed tree-based strategy is particularly successful when applied in an ensemble model, as shown by the fact that the relative efficiency is even higher in the random forest model.

### Case 2

Duration of delivery is a unique experience. Sometimes it’s over in a matter of hours. Delivery duration is the time of procedure that will give birth to your child [27]. As delivery duration is also an important variable which must be studied so in in Case 2, the usage of iron tablets during pregnancy has been taken as stratum-specific variable and the duration of delivery has been taken as study variable.

Table 4 shows bootstrap study results for case 2 i.e., “delivery duration” as the study variable and “usage of iron tablets” as stratum membership variable. There are five different CART models according to tree parameters and one random forest (*rf*) model. ${\hat{\lambda }}_{h}$ shows the stratum proportion for strata i.e., *h* = 1, *and h* = 2. ${\hat{\mu }}_{yh}$ represents the mean of respected stratum.

**Table 4:** Bootstrap results Case 2.

		Stratum 1					Stratum 2				
n	Models	$\hat{\lambda_{h}}$	${\hat{\mu }}_{yh}$	EAPE	MSPE	RE	$\hat{\lambda_{h}}$	${\hat{\mu }}_{yh}$	EAPE	MSPE	RE
**20**	**1**	0.4958	2.8608	0.1358	0.5089	1.3611	0.5051	2.9624	0.0253	0.4943	1.4033
	**2**	0.4958	2.9272	0.0694	0.4700	1.4738	0.5051	2.9045	0.0325	0.4607	1.5058
	**3**	0.4844	2.9282	0.0684	0.4687	1.4778	0.5152	2.9068	0.0302	0.4591	1.5111
	**4**	0.4958	2.8601	0.1359	0.5092	1.3604	0.5051	2.9633	0.0262	0.4954	1.4002
	**5**	0.4844	2.8618	0.1348	0.5078	1.3643	0.5152	2.9655	0.0284	0.4942	1.4037
	**rf**	0.4546	2.9119	0.0846	0.4104	1.6877	0.5380	2.9128	0.0242	0.4063	1.7075
**30**	**1**	0.5013	2.8551	0.2339	0.3671	1.3977	0.4987	2.9387	0.0263	0.3104	1.4625
	**2**	0.5013	1.9152	0.1739	0.3297	1.5561	0.4987	2.8939	0.0711	0.2954	1.5370
	**3**	0.4908	2.9163	0.1727	0.3283	1.5631	0.5065	2.8965	0.0685	0.2937	1.5458
	**4**	0.5013	2.8553	0.2336	0.3681	1.3940	0.4987	2.9400	0.0250	0.3129	1.4511
	**5**	0.4908	2.8564	0.2326	0.3666	1.3997	0.5067	2.9425	0.0225	0.3116	1.4568
	**rf**	0.4529	2.9115	0.1775	0.2867	1.7895	0.5410	2.8968	0.0681	0.2532	1.7929
**40**	**1**	0.5025	2.8492	0.3380	0.3481	1.3685	0.4971	2.9267	0.0958	0.2459	1.4384
	**2**	0.5025	2.9014	0.2858	0.3063	1.5552	0.4971	2.8901	0.1325	0.2414	1.4652
	**3**	0.4930	2.9030	0.2842	0.3050	1.5619	0.5032	2.8927	0.1299	0.2406	1.4698
	**4**	0.5025	2.8469	0.3403	0.3511	1.3568	0.4971	2.9270	0.0955	0.2487	1.4223
	**5**	0.4930	2.8491	0.3381	0.3496	1.3626	0.5032	2.9292	0.0933	0.2481	1.4254
	**rf**	0.4474	2.9039	0.2833	0.2739	1.7393	0.5466	2.8934	0.1292	0.2103	1.6816

Table 4 shows that the mean estimated time for delivery is almost equal in both strata i.e., stratum 1 and stratum 2. The relative efficiency is greater than all other single tree models in all results because random forest always provide good results as compared to single trees as the number of trees are more than 1 i.e., 500 in random forest.

We assessed 5 classification and regression models and 1 random forest model in our study and determined the EAPE for each model. No significant change is observed in EAPE values with change in tree parameters, however EAPE values have a slightly increasing trend with increase in sample size. Further, EAPE values are higher in smaller stratum (*h* = 1) as compared to the larger one (*h* = 2).

**Fig 4:**
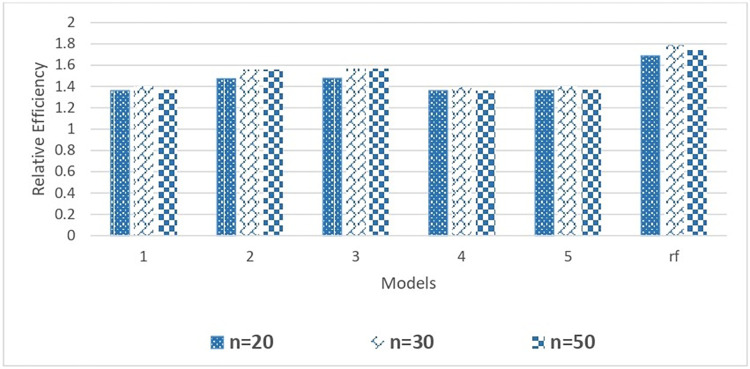
Comparison of relative efficiency of the mean estimator with different DGCART models for different sample sizes under Case 2.

**Fig 5:**
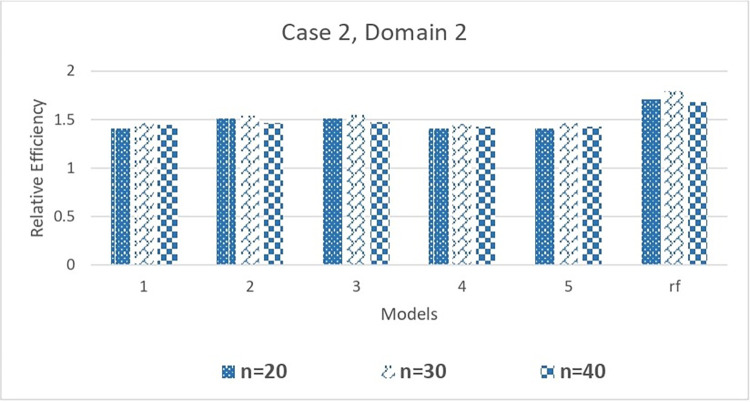
Comparison of relative efficiency of the mean estimator with different DGCART models for different sample sizes under Case 2.

Table 4 also provided the Mean Squared Prediction Error for 5 single classification and regression tree models and 1 random forest model. Similar to EAPE, there is no significant change in MSPE values with change in tree parameters. However, the relative MSPE values corresponding to random forest is significantly smaller than all other single tree models. The relative efficiency (RE) values are greater than one for all combinations of tree parameters showing superiority of tree-based total estimators to corresponding estimators without utilizing any tree. The results given in Table 4 can be visualized from Figs 4 and 5.

Fig 4 provides a graphical representation the relative efficiency of the mean estimator when delivery duration is used as the study variable. For n = 20, 30 and 50, we have 5 single CART models and 1 random forest model. All of the models’ relative efficiency patterns are more than 1.20, with the random forest models’ pattern exceeding 1.65. In comparison to larger sample sizes, the relative efficiency for all single CART and random forest models is relatively low for n = 20.

Fig 5 illustrates the relative efficacy of mean estimator when delivery time is used as a variable of interest. The random forest model is more efficient than all single CART models, with a relative efficiency of more than 1.40. It is simple to determine that the relative efficiency varies in accordance with the hyper-parameter values in each single CART model.

As evidenced by its relative efficiency being higher in the random forest model and greater than 1 in all single tree models, our proposed tree-based method is more effective than the existing method. A value larger than 1 shows that the proposed technique is more efficient. Relative efficiency assesses the enhancement in efficiency of the proposed method over the existing method From both figures and tables we infer that the simultaneous application of classification and regression tree for stratification of non-sampled units assist in efficiency improvement when appropriate hype-parameters for trees are set for the training task. Ensemble different trees for the said two tasks provide the best performance of the total estimator for the variable of interest in different domains.

## 5. Conclusion

This study focused on classifying the non-sampled units into different strata using a classification tree algorithm and predicting the value of the study variable for the unobserved part of the population using a regression tree algorithm. Due to their ease of interpretation, and visualization tree-based algorithms are considered good alternatives to classical regression and classification models. The tree-based algorithms also deal with prediction and classification problems when the parametric relationship between the study variable and the predictors is ambiguous. Due to these attractive features, the tree DGCART method is proposed for estimating stratum-specific parameters. With random forest decision trees one can make predictions from different random samples of covariates rather than selecting the best ones and enhance the precision of the estimators proposed. Bagging in random forests also provides a direct estimate of prediction variance that can be considered in future studies. Similar studies, where stratum-specific estimates are needed, can benefit from the current study’s representation of how various input factors might be used to forecast a target value and utilized in the estimation stage.

The DGCART algorithm is especially useful in obtaining estimates of different indicators in specific demographic, socio-economic and geographic subpopulations in health related surveys where the indicator of interest has a high proportion of missing observations. Where missing part of the actual sample can be considered as the non-sampled part.
