# Correction: Stratum-specific health outcome estimation in Pakistan using double goal CART

**DOI:** 10.1371/journal.pone.0321510

**Published:** 2025-04-01

**Authors:** Muhammad Hamza, Shakeel Ahmed

There are errors in the Author Contributions. The correct contributions are:

**Conceptualization:** Shakeel Ahmed

**Data curation:** Muhammad Hamza, Shakeel Ahmed

**Formal analysis:** Muhammad Hamza

**Investigation:** Muhammad Hamza, Shakeel Ahmed

**Methodology:** Muhammad Hamza, Shakeel Ahmed

**Resources:** Muhammad Hamza, Shakeel Ahmed

**Supervision:** Shakeel Ahmed

**Validation:** Shakeel Ahmed

**Visualization:** Muhammad Hamza

**Writing – original draft:** Muhammad Hamza

**Writing – review & editing:** Muhammad Hamza, Shakeel Ahmed
